# Is Homocysteine Associated with the Prognosis of Covid-19 Pneumonia

**DOI:** 10.1155/2023/9697871

**Published:** 2023-03-02

**Authors:** Işılay Kalan Sarı, Olgun Keskin, Ayşegül Seremet Keskin, Hamit Yaşar Elli̇dağ, Orbay Harmandar

**Affiliations:** ^1^Department of Endocrinology and Metabolic Disorders, University of Health Sciences, Antalya Training and Research Hospital, Antalya, Turkey; ^2^Department of Chest Disease, University of Health Sciences, Antalya Training and Research Hospital, Antalya, Turkey; ^3^Department of Infectious Disease and Clinical Microbiology, University of Health Sciences, Antalya Training and Research Hospital, Antalya, Turkey; ^4^Department of Clinic of Biochemistry, University of Health Sciences, Antalya Training and Research Hospital, Antalya, Turkey; ^5^Department of Critical Care, University of Health Sciences, Antalya Training and Research Hospital, Antalya, Turkey

## Abstract

*Background*/*Aim*. Coronavirus disease 2019 (COVID-19) is a life-threatening disease characterized by a prothrombotic state. Because homocysteine (Hcy) is a potential biomarker in thrombotic disease, this article aims to highlight the role of Hcy in the prognosis of COVID-19 pneumonia. *Methods*. This prospective study was conducted between April 2021 and December 2021 at the University of Health Sciences, Antalya Training and Research Hospital. 162 patients admitted to the emergency department for COVID-19 pneumonia and scheduled for hospitalization in the intensive care unit (ICU) or COVID-19 ward of the chest disease department were included in the study. Hcy levels and other necessary laboratory parameters were analyzed. *Results*. 134 patients were admitted to the COVID-19 ward and 28 to the ICU. Hcy levels were significantly higher in ICU patients than in ward patients (*p* : 0.001). Of the 134 patients, 55 later required ICU treatment for various reasons and were transferred to the ICU. Hcy (*p* : 0.010), ferritin (*p* : 0.041), and LDH (*p* : 0.010) were significantly higher in patients who were transferred to the ICU than in patients who remained in the ward. The Hcy level was associated with a poor prognosis. It was found that each unit increase in the Hcy level approximately doubled the risk of death in patients with COVID-19 (odds ratio: 1.753). *Discussion*. There are few studies examining the association between high Hcy levels and disease severity in COVID-19. Our study supports previous studies and shows the association between the need for intensive care and high Hcy levels. *Conclusion*. A high Hcy value is a helpful marker in determining the need for critical care on admission to the emergency department and a marker of poor prognosis in COVID-19 pneumonia.

## 1. Introduction

Since the 2019 coronavirus (COVID-19) pandemic, reliable biomarkers associated with the disease prognosis are needed for early identification of high-risk patients. Identification of new biomarkers helps to categorize patients immediately after diagnosis, plan hospitalization, and improve clinical management of the disease. COVID-19 infection is associated with coagulopathy, and there is an increase in procoagulant factors such as fibrinogen and D-dimer [1, 2]. The incidence of pulmonary embolism is increased [3], and microvascular thrombosis in the lungs is demonstrated in the autopsy series and in COVID-19 acute respiratory distress syndrome [4–6]. Leukocytes activated during the inflammatory storm involving IL-6 may damage the endothelium in capillaries and impair the thromboprotective mechanism of the endothelial cells. On the other hand, the virus can infect cells via the angiotensin-converting enzyme-2 receptor, which is mainly expressed on endothelial cells and cause vascular damage [7–10]. This endotheliitis may be the cause of this microthrombotic disorder, which particularly affects the lungs and kidneys [8, 11]. In addition, recent studies have shown that homocysteine (Hcy) may be associated with this microvascular thrombosis and the severity of COVID-19 disease [12–14]. Hcy is associated with pulmonary and venous thrombosis, and high plasma Hcy levels significantly increase the incidence of vascular injury in both small and large vessels [15–17]. Concentrations above the 90th percentile are associated with an increased risk of degenerative and atherosclerotic processes in the coronary, cerebral, and peripheral circulatory systems [18]. Although Hcy is an effective cardiovascular risk biomarker and cardiovascular complications are considered crucial in hospitalized COVID-19 patients, there are few detailed studies on this parameter. The aim of our study was to compare Hcy levels in patients admitted to the emergency department for COVID-19 pneumonia and scheduled for inpatient admission to the intensive care unit (ICU) or COVID-19 ward and to demonstrate the association between Hcy levels and disease prognosis.

## 2. Methods

Our study was designed as a prospective cross-sectional study. The study was approved by the Ethics Committee of the University of Health Sciences, Antalya Training and Research Hospital (date: 18/03/2021, number: 3/22), and written informed consent was obtained from all patients/guardians. The report was prepared in accordance with the Declaration of Helsinki. This study was conducted between April 2021 and December 2021 at the University of Health Sciences, Antalya Training and Research Hospital, COVID-19 ward in the chest disease unit and the ICU. A total of 162 patients who were admitted to the emergency department and had an indication for hospitalization in the ICU or COVID-19 ward due to COVID-19 pneumonia were included. Patients were excluded if they were younger than 18 years and older than 75 years; had a history of malignancy, coronary artery disease or valvular heart disease, liver or kidney failure, chronic inflammatory disease, and thromboembolic disease; were taking antiplatelet or anticoagulant medications; and were pregnant or breastfeeding. The diagnosis of COVID-19 infection was made from nasopharyngeal swabs that were positive in the reverse transcription-polymerase chain reaction. The diagnosis of pneumonia was made by thoracic computed tomography (CT), laboratory tests, and clinical evaluation. The indications for hospitalization in the ICU and COVID-19 ward of the patients were determined according to the treatment guidelines of the Turkish Ministry of Health regarding COVID-19 for adult patients [19]. Within the first 2 hours after hospitalization, blood samples were taken from the patients for necessary biochemical and hematological tests and were studied in our laboratory. The biochemical tests, including BUN (blood urea nitrogen), creatinine, albumin, ALT (alanine aminotransferase), AST (aspartate aminotransferase), LDH (Lactate dehydrogenase enzyme), ferritin, total cholesterol (TC), triglycerides (TG), HDL-C (high-density lipoprotein-cholesterol), and CRP (C-reactive protein), were analyzed by the spectrophotometric method using Beckman Coulter commercial kits in the Beckman Coulter AU5800 (Beckman Coulter Inc. CA, USA) autoanalyzer. LDL-C (low-density lipoprotein-cholesterol) was calculated according to the Friedewald formula [20]. Hemogram (whole blood analysis) was analyzed on Sysmex XT-2000i (Sysmex, Kobe, Japan). Fibrinogen and D-dimer assays were performed on the ACL-TOP 700 (Instrumentation Laboratory, Bedford, Massachusetts, USA) coagulation autoanalyzer using ACL Synthesis commercial kits. After performing these tests, blood samples were centrifuged at 3500 rpm for 10 minutes. The separated serums were stored in sealed 1.5 ml Eppendorf tubes at −80°C for later assay to measure the serum Hcy level. The separated serums were thawed at room temperature on the study day to determine the Hcy levels. Human Hcy was analyzed using ELISA commercial kits (AFG Bioscience, Illinois, United States, intraassay reproducibility (precision within an assay) <8%, interassay reproducibility (precision between assays) <10%, detection range: 0.75–15 *µ*mol/L, sensitivity: 0.3 *µ*mol/L). Treatment of patients was started in accordance with the COVID-19 treatment guidelines of the Turkish Ministry of Health when patients were enrolled in the study. Patients received 10 days of favipiravir therapy, low-molecular-weight heparin, and prednisolone according to the degree of pneumonia. Patients were followed up during hospitalization.

## 3. Statistical Analysis

The SPSS 23.0 program was used for the analysis. Conformity of variables to the normal distribution was examined using visual (histogram and probability graphs) and analytical methods (Kolmogorov–Smirnov/Shapiro–Wilk tests). Descriptive analyzes are performed using the mean and standard deviations for normally distributed variables. Means and standard deviations of patients' age and biochemical data were reported, and the difference between groups was analyzed with Student's *t*-test. Differences between categorical variables were evaluated with the chi-square test. Whether the parameters were normally distributed or not, Student's *t*-test and the Mann–Whitney *U* test were used to evaluate the biochemical data of the patients according to the poor prognosis status. The statistical significance level was set as *p* value <0.05. The effects of some biochemical findings of patients on the disease prognosis and mortality risk were evaluated by the logistic regression analysis. Decision-making of Hcy levels in predicting the probability of exitus was analyzed by the receiver operating characteristics (ROCs) curve. Sensitivity and specificity values of the significant cut-off value were calculated. In the evaluation of the area under the curve, it was considered to be statistically significant in cases where the type-1 error level was below 5%.

## 4. Results

**Figure 1:**
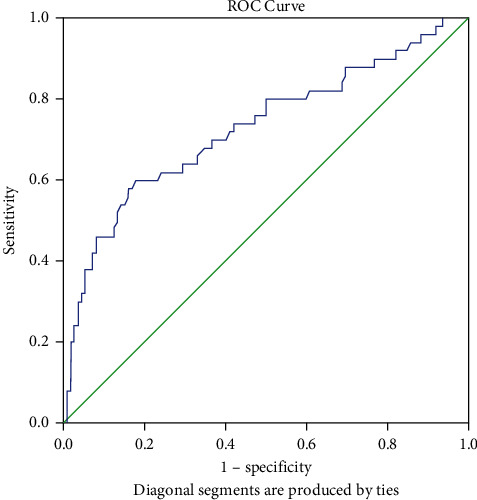
Receiver operating characteristics (ROCs) curve of homocysteine levels in predicting the probability of patients exitus (developing a poor prognosis).

According to the current clinical status of 162 patients enrolled in the study, 134 (82.7%) were admitted from the emergency department to the COVID-19 ward and 28 (17.3%) were admitted to the ICU. Patients in the ICU and in the COVID-19 ward were compared (Table 1). Regarding admission symptoms, nausea/vomiting occurred more frequently in patients in the COVID-19 ward (*p* : 0.005). SpO_2_ (oxygen saturation) levels at the time of admission were comparable between the 2 groups (*p* : 0.073). The length of hospital stay was longer in ICU patients than in ward patients (*p* : 0.0001). Laboratory results of patients in the ICU and in the COVID-19 ward were compared (Table 2). It was found that albumin levels were lower in ICU patients than in ward patients (*p* : 0.011). Hcy levels were significantly higher in ICU patients than in ward patients (*p* : 0.001). Of the 134 patients admitted to the COVID-19 ward on admission, 55 of them later developed a need for ICU care for various reasons and were transferred to the ICU. Hcy (*p* : 0.010), ferritin (*p* : 0.041), and LDH (*p* : 0.010) were significantly higher in patients who were transferred to the ICU than in patients who remained in the ward. There were 61 patients with a hospital stay of less than 10 days and 101 patients with a hospital stay of more than 10 days. When comparing patients with hospitalization of more than 10 days and less than 10 days, fibrinogen (*p* : 0.019), ferritin (*p* : 0.021), CRP (*p* : 0.016), and LDH (*p* : 0.004) were higher in patients with hospitalization of more than 10 days while lymphocyte count (*p* : 0.002) was lower. It was found that 112 patients were discharged and 50 were exitus. The values of Hcy (*p* : 0.0001), D-dimer (*p* : 0.003), N/L ratio (*p* : 0.019), CRP (*p* : 0.0001), and LDH (*p* : 0.016) were significantly higher in exitus patients compared to discharged patients. The comparison of laboratory values between groups according to the prognosis status is shown in Table 3. We evaluated the effects of some biochemical markers known to be important for the prognosis of COVID-19 and Hcy on the prognosis of the disease (depending on whether they are exitus or not) by the logistic regression analysis. It was found that each unit by which Hcy increased approximately doubled the risk of death in patients with COVID-19 (odds ratio: 1.753) (Table 4). When the cut-off value of Hcy in predicting ICU admission was taken as 5.85, the sensitivity was found to be 66% and the specificity as 67% (Table 5). The ROC curve is given in Figure 1. Considering the correlation between Hcy and other parameters, Hcy was positively related with D-dimer and platelets count (*p* : 0.01 and *p* : 0.19, respectively) (Table 6).

## 5. Discussion

It is known that the severity and prognosis of COVID-19 disease are different in each patient. It has been shown that 10–15% of mild cases have a severe course and 15–20% of severe cases may require intensive treatment [21]. In our study, 17% (28 patients) of 162 patients admitted to the emergency department had an indication for admission to the ICU. During the follow-up of 134 patients in the ward, 44% (55 individuals) of them required intensive care for various reasons. Numerous biomarkers continue to be studied to predict the disease prognosis and identify patients who may require intensive care. Studies have shown that lymphopenia, low platelet counts, and a high N/L ratio are related to disease severity [22]. Liver tests indicative of liver injury, particularly ALT, ferritin, CRP levels, and D-dimer, have been shown to be associated with a poor prognosis and mortality, and a decrease in albumin levels also indicates a poor prognosis [22]. Our study shows that low albumin and high Hcy levels may be indicators of which patients should be transferred to the ICU on admission to emergency department. It is also evident from our study that these two parameters are related to the severity of the disease. The parameters associated with survival in our study were Hcy, D-dimer, N/L ratio, CRP, and LDH. It was found that each unit by which Hcy increased approximately doubled the risk of death in patients with COVID-19. Hcy was associated with poor prognostic parameters, including exitus, and need for intensive care. According to recent studies, people with COVID-19 have a significantly higher risk of developing coagulopathy and thrombosis, two conditions in which D-dimer levels rise, especially in severe courses. It is thought that the amino acid Hcy, which is essential for coagulation, may potentially be a factor in these conditions [14]. As an indicator of the likelihood of unfavorable disease progression in COVID-19 patients, elevated Hcy levels appear to be beneficial. In the study by Yang et al. involving 273 COVID-19 patients, Hcy levels were measured and chest CT was performed on hospital admission. They performed CT on day 7 ± 2 of admission to assess disease progression during the first week. Hcy levels were significantly higher on imaging in patients with disease progression than in patients without progression [12]. Ponti et al. measured plasma Hcy levels in 304 hospitalized COVID-19 patients and found that serum Hcy levels were significantly higher in survivors than in exitus patients [13]. Keskin et al. treated 117 hospitalized COVID-19 patients, and Hcy levels were higher in the severe disease group (respiratory rate above 30/min, blood oxygen saturation below 93%, respiratory failure, shock, or other organ failure requiring intensive care) than in the mild disease group (respiratory rate below 30/min and blood oxygen saturation above 90% on room air) [23]. In the study by Fouda et al. of 40 pediatric patients with COVID-19, the Hcy levels were higher in patients than in controls and were associated with disease severity and need for intensive care [24]. There are few studies investigating the association between high Hcy levels and disease severity in COVID-19 [25]. Our study supports these studies and demonstrates the association between the need for intensive treatment and high Hcy levels. A limitation was the small number of patients receiving intensive treatment at the time of hospitalization. Patients in the study received similar treatments. Before starting the treatment, the blood drawn was centrifuged and stored, and Hcy measurements were obtained from these serums. Therefore, the treatment had no effect on Hcy levels.

## 6. Conclusion

A high Hcy level is a helpful marker in determining the need for intensive care on admission to the emergency department and a marker of poor prognosis in COVID-19 pneumonia. Measurement of Hcy in patients admitted to the hospital with COVID-19 pneumonia is beneficial for early identification of patients with a poor prognosis and for predicting the need for intensive care. Identifying patients who have a poor prognosis at the time of admission and appropriately predicting the need for hospitalization in the ICU may help to improve early management of these patients in the ICU and reduce mortality.

## Figures and Tables

**Table 1:** Evaluation of demographic characteristics, concomitant symptoms, and clinical findings of patients admitted to the COVID-19 ward and intensive care unit.

	COVID-19 ward (*n* : 134)	Intensive care unit (*n* : 28)	*p*
*Gender (n* : *%)*			
Male	82 (61.2)	19 (67.9)	*x* ^2^: 0.438; *p* : 0.669
Female	52 (38.8)	9 (32.1)	
Age (years) (*x* ± SD)	60.8 ± 13.91	62.1 ± 13.65	0.643
Length of hospital stay (day) (*x* ± SD)	14.5 ± 9.89	25.0 ± 24.14	0.0001^*∗*^

*Accompanying Symptoms and Clinical Findings (n* : *%)*			
Fever	41 (30.6)	5 (17.9)	*x* ^2^: 1.849; *p* : 0.249
Cough	65 (48.5)	9 (32.1)	*x* ^2^: 2.500; *p* : 0.145
Dyspnea	77 (57.5)	18 (64.3)	*x* ^2^: 0.445; *p* : 0.535
Loss of taste and smell	4 (3.0)	—	*x* ^2^: 0.857; *p* : 1.000
Diarrhea	6 (4.5)	—	*x* ^2^: 1.302; *p* : 0.591
Sputum	9 (6.7)	4 (14.3)	*x* ^2^: 1.798; *p* : 0.242
Nausea/vomiting	28 (20.9)	—	*x* ^2^: 7.073; *p* : 0.005^*∗*^
Chest pain	6 (4.5)	2 (7.1)	*x* ^2^: 0.350; *p* : 0.628
Back pain	5 (3.7)	1 (3.6)	*x* ^2^: 0.002; *p* : 1.000
Muscle joint pain	17 (12.7)	1 (3.6)	*x* ^2^: 1.948 *p* : 0.317
SpO_2_ (%) (*x* ± SD)	92.0 ± 6.38	89.6 ± 6.27	0.073
Temperature (°C) (*x* ± SD)	37.0 ± 0.77	36.9 ± 0.53	0.637

^*∗*^ *p* < 0.05 SpO_2_: oxygen saturation.

**Table 2:** Laboratory results of patients with COVID-19 admitted to the COVID-19 ward and intensive care unit.

Laboratory results	COVID-19 ward (*n* : 134)	Intensive care unit (*n* : 28)	*p*
	(*x* ± SD)	(*x* ± SD)	
BUN (mg/dL)	24.0 ± 16.09	30.8 ± 22.43	0.060
Cre (mg/dL)	1.0 ± 0.65	1.1 ± 0.59	0.692
Alb (g/dL)	3.6 ± 2.78	2.9 ± 0.59	0.011^*∗*^
ALT (U/L)	45.3 ± 41.3	53.4 ± 35.41	0.337
AST (U/L)	45.7 ± 38.50	48.1 ± 27.57	0.767
LDH (U/L)	400.3 ± 211.60	486.4 ± 246.47	0.079
TC (mg/dL)	166.3 ± 53.13	137.5 ± 51.01	0.130
HDL (mg/dL)	40.4 ± 11.29	33.5 ± 13.20	0.106
LDL (mg/dL)	88.6 ± 39.1	76.5 ± 37.30	0.386
TG (mg/dL)	170.4 ± 97.48	152.7 ± 65.52	0.447
CRP (mg/L)	78.1 ± 71.65	96.1 ± 77.50	0.240
WBC (10^3^/mm^3^)	9.5 ± 5.01	10.1 ± 4.28	0.646
HGB (g/dL)	12.5 ± 1.96	12.1 ± 2.37	0.409
PLT (10^3^/mm^3^)	249.9 ± 125.48	242.8 ± 134.08	0.289
Lymphocyte (%)	12.9 ± 11.38	11.8 ± 14.78	0.654
N/L ratio	15.1 ± 33.10	16.2 ± 14.86	0.852
Ferritin (*µ*g/L)	627.6 ± 1092.80	762.9 ± 833.97	0.578
D-dimer (*µ*g/L)	1165.9 ± 2301.51	2175.2 ± 2872.93	0.056
Fibrinogen (mg/dL)	487.9 ± 168.12	556.1 ± 243.23	0.101
Hcy (*µ*mol/L)	5.8 ± 1.57	7.10 ± 2.12	0.001^*∗*^

^*∗*^ *p* < 0.05. BUN, blood urea nitrogen; Cre, creatinine; Alb, albumin; ALT, alanine aminotransferase; AST, aspartate aminotransferase; LDH, lactate dehydrogenase enzyme; TC, total cholesterol; TG, triglycerides; HDL-C, high-density lipoprotein-cholesterol; LDL-C, low-density lipoprotein-cholesterol; Hcy, homocysteine; CRP, C-reactive protein; WBC, white blood cells; HGB, hemoglobin; PLT, platelet; N/L, neutrophil-to-lymphocyte ratio.

**Table 3:** Evaluation of laboratory results according to the prognosis status of the patients.

Laboratory results	Exitus (*n* : 50)	Discharged (*n* : 112)	*p*	Length of stay <10 days (*n* : 61)	Length of stay ≥10 days (*n* : 101)	*p*	Continue to be hospitalized in the COVID-19 ward (*n* : 79)	Transferred from the service to the intensive care unit (*n* : 55)	*p*
BUN (mg/dL)	31.5 ± 20.32	22.3 ± 15.27	0.002^*∗*^	22.4 ± 15.57	26.9 ± 18.41	0.118	22.5 ± 16.72	25.9 ± 15.12	0.240
Cre (mg/dL)	1.1 ± 0.60	1.0 ± 0.65	0.417	1.0 ± 0.47	1.1 ± 0.72	0.506	1.0 ± 0.55	1.2 ± 0.76	0.202
Alb (g/dL)	2.9 ± 0.55	3.7 ± 3.00	0.091	3.5 ± 0.65	3.4 ± 3.23	0.964	3.4 ± 0.66	3.9 ± 4.16	0.378
ALT (U/L)	46.9 ± 34.62	46.6 ± 43.10	0.972	46.0 ± 40.95	47.2 ± 40.47	0.867	44.2 ± 41.04	46.9 ± 42.15	0.719
AST (U/L)	44.0 ± 23.15	47.0 ± 41.43	0.658	46.2 ± 47.52	46.0 ± 28.99	0.976	41.7 ± 38.33	51.0 ± 38.43	0.187
LDH (U/L)	482.0 ± 206.40	386.1 ± 219.15	0.016^*∗*^	347.9 ± 237.60	454.2 ± 198.37	0.004^*∗*^	358.4 ± 171.39	457.9 ± 247.12	0.010^*∗*^
TC (mg/dL)	156.6 ± 46.05	159.5 ± 58.89	0.869	166.1 ± 51.45	155.4 ± 54.86	0.579	175.3 ± 51.75	155.2 ± 54.73	0.319
HDL (mg/dL)	40.5 ± 13.81	37.3 ± 10.93	0.409	38.8 ± 9.75	38.5 ± 13.07	0.956	40.4 ± 11.95	40.5 ± 10.84	0.991
LDL (mg/dL)	87.6 ± 37.32	83.3 ± 40.15	0.735	82.1 ± 22.14	86.1 ± 42.63	0.790	90.9 ± 33.67	86.2 ± 45.6	0.762
TG (mg/dL)	161.2 ± 81.68	168.8 ± 96.52	0.721	178.5 ± 120.59	160.6 ± 76.35	0.458	186.5 ± 106.76	155.5 ± 87.40	0.256
CRP (mg/L)	111.7 ± 74.34	67.9 ± 68.24	0.0001^*∗*^	63.6 ± 73.79	91.9 ± 70.29	0.016^*∗*^	69.5 ± 71.71	90.2 ± 70.42	0.101
WBC (10^3^/mm^3^)	10.6 ± 6.23	9.23 ± 4.10	0.084	9.5 ± 5.47	9.8 ± 4.52	0.724	9.6 ± 5.21	9.5 ± 4.77	0.940
HGB (g/dL)	12.1 ± 2.31	12.5 ± 1.90	0.247	12.3 ± 1.90	12.6 ± 2.11	0.386	12.4 ± 2.08	12.5 ± 1.79	0.732
PLT (10^3^/mm^3^)	236.1 ± 140.83	254.3 ± 119.96	0.399	257.5 ± 135.15	243.4 ± 121.54	0.492	258.8 ± 125.26	237.5 ± 125.86	0.334
Lymphocyte (%)	10.4 ± 12.03	13.8 ± 11.87	0.097	16.3 ± 14.14	10.5 ± 9.92	0.002^*∗*^	13.3 ± 11.05	12.3 ± 11.90	0.610
N/L ratio	23.7 ± 52.24	11.5 ± 10.76	0.019^*∗*^	11.0 ± 12.52	17.8 ± 37.50	0.099	12.1 ± 11.71	19.1 ± 49.29	0.233
Ferritin (*µ*g/L)	746.7 ± 645.83	611.5 ± 1181.94	0.509	422.2 ± 436.11	784.5 ± 1258.19	0.021^*∗*^	422.6 ± 401.86	862.4 ± 1516.81	0.041^*∗*^
D-dimer (*µ*g/L)	2198.6 ± 3127.27	948.1 ± 1934.04	0.003^*∗*^	970.2 ± 1847.05	1547.1 ± 2969.85	0.118	1209.4 ± 2503.88	1105.6 ± 2008.16	0.802
Fibrinogen (mg/dL)	540.0 ± 188.78	480.3 ± 177.78	0.068	454.1 ± 157.42	524.2 ± 191.82	0.019^*∗*^	473.2 ± 169.20	508.7 ± 166.00	0.250
Hcy (*µ*mol/L)	7.0 ± 2.01	5.64 ± 1.39	0.0001^*∗*^	5.7 ± 1.70	6.3 ± 1.73	0.072	5.5 ± 1.48	6.3 ± 1.61	0.010^*∗*^

^*∗*^ *p* < 0.05. BUN, blood urea nitrogen; Cre, creatinine; Alb, albumin; ALT, alanine aminotransferase; AST, aspartate aminotransferase; LDH, lactate dehydrogenase enzyme; TC, total cholesterol; TG, triglycerides; HDL-C, high-density lipoprotein-cholesterol; LDL-C, low-density lipoprotein-cholesterol; Hcy, homocysteine; CRP, C-reactive protein; WBC, white blood cells; HGB, hemoglobin; PLT, platelet; N/L, neutrophil-to-lymphocyte ratio.

**Table 4:** Evaluation of the effects of some biochemical findings of the patients on the prognosis of the disease (according to whether they are exitus or not) by logistic regression analysis.

	B	S.E	*p*	Beta (OR)	95% C.I. for EXP (B)	
					Lower	Upper
Alb	−0.324	0.449	0.471	0.723	0.300	1.744
LDH	0.001	0.001	0.452	1.001	0.999	1.003
CRP	0.006	0.003	0.070	1.006	1.000	1.012
Lymphocyte	−0.013	0.030	0.679	0.988	0.931	1.048
N/L ratio	0.012	0.011	0.301	1.012	0.989	1.035
D-dimer	0.000	0.000	0.568	1.000	1.000	1.000
Hcy	0.561	0.156	0.0001^∗^	1.753	1.292	2.378

Alb, albumin; LDH, lactate dehydrogenase enzyme; CRP, C-reactive protein; N/L, neutrophil-to-lymphocyte ratio; Hcy, homocysteine; OR, odds ratio ^*∗*^*p* < 0.05.

**Table 5:** Evaluation of patients' homocysteine levels in predicting the possibility of exitus (developing a poor prognosis).

Risk factor	AUC (%95)	Cut off	*p*	Sensitivity (%)	Specificity (%)
Hcy	0.729 (0.638–0.819)	5.85	0.0001^∗^	66.0	67.0

Hcy, homocysteine; AUC, area under the curve ^*∗*^*p* < 0.05.

**Table 6:** The relationship between homocysteine levels and other biochemical parameters.

		FBN	D-DM	FER	N/L	LN	BUN	CRE	ALB	ALT	AST	LDH	TC	HDL	LDL	TG	CRP	WBC	HGB	PLT
Hcy	*r*	0.014	0.256	−0.011	0.055	−0.098	0.124	0.010	−0.105	0.060	−0.071	0.140	−0.098	−0.134	−0.134	0.060	0.092	0.138	−0.089	0.182
	*p*	0.865	0.001^*∗*^	0.903	0.490	0.215	0.116	0.896	0.196	0.453	0.395	0.094	0.547	0.403	0.422	0.614	0.249	0.080	0.263	0.019^*∗*^

^*∗*^ *p* < 0.05. FBN, fibrinogen; D-DM, D-dimer; FER, ferritin; N/L, neutrophil-to-lymphocyte ratio; LN, lymphocyte; BUN, blood urea nitrogen; CRE, creatinine; ALB, albumin; ALT, alanine aminotransferase; AST, aspartate aminotransferase; LDH, lactate dehydrogenase enzyme; TC, total cholesterol; TG, triglycerides; HDL-C, high-density lipoprotein-cholesterol; LDL-C, low-density lipoprotein-cholesterol; Hcy, homocysteine; CRP, C-reactive protein; WBC, white blood cells; HGB, hemoglobin; PLT, platelet.

## Data Availability

Data analyzed during the current study are available from the corresponding author upon request.

## Additional Points

*What is Known? What is New?.* A limited number of previous studies have found that homocysteine may be associated with poor prognosis in COVID-19 and that it is higher in severe disease than in mild disease. However, in our study, we found that the measurement of homocysteine is a guide for early decision-making about which patients need intensive care on initial admission to the emergency department, and we also showed that it is directly related to mortality and is a useful tool for early identification of patients who may need intensive care.
